# Psychosocial and Emotional Wellbeing of Older Adults With HIV

**DOI:** 10.1093/geroni/igaf122.940

**Published:** 2025-12-31

**Authors:** Anna Rubtsova, Paul Nash, David Vance

## Abstract

In the era of modern antiretroviral therapy (ART) when treated people with HIV enjoy improved health outcomes and live nearly normal lifespans, psychosocial and emotional wellbeing of older people with HIV (OPWH) increasingly comes into focus. First, studies are emerging that show that in OPWH with well-controlled HIV important health outcomes, such as aging-related comorbidities, may be less related to HIV and increasingly associated with social and psychosocial determinants of health. Second, there is a growing realization that quality of life, emotional wellbeing, and mental health are important outcomes for whole-person care of OPWH. This symposium explores both these themes. Our first presentation is based on a scoping review of published research examining social network structure, social support, and isolation among older women with HIV and highlights the associations between social relationships and wellbeing. The second presentation discusses results of a qualitative study and explores emotional wellbeing and trust among older adults relative to younger adults with HIV during COVID-19 pandemic in New York City. Our third presentation showcases significant associations between perceived stress, loneliness, social support and frailty in women with HIV, based on statistical analysis of data by the Women’s Interagency HIV Study (WIHS). Lastly, our final presentation focuses on loneliness and social engagement as targets for public health interventions in OPWH, reporting results related to the development and pilot testing of B’ACTIV behavioral activation intervention. Together, these presentations provide a broad perspective on ongoing research and future directions for exploring psychosocial and emotional wellbeing in OPWH. HIV, AIDS and Older Adults Interest Group Sponsored Symposium

